# Topiramate-induced ocular complications: case series

**DOI:** 10.22336/rjo.2024.14

**Published:** 2024

**Authors:** Sarita Lobo, Geover Joslen Lobo

**Affiliations:** * Department of Ophthalmology, Father Muller Medical College, Mangalore, India

**Keywords:** Topiramate, myopic shift, acute angle closure, migraine prophylaxis

## Abstract

Several ocular adverse effects have been attributed to Topiramate, a sulfonamide derivative. It can cause problems in the eye such as choroidal effusion syndrome, acute angle closure glaucoma, myopic shift, visual field defects, and Myokymia. If not identified early, it can be vision-threatening.

It is commonly used for migraine prophylaxis, partial onset, and generalized tonic-clonic seizures. It has also been prescribed for bipolar disorder and alcoholism. The risk of adverse reactions with this drug is 3%. The prognosis is favorable if it is discontinued early and prompt therapy is initiated.

Objective: This article reported a case series of topiramate-induced ocular complications.

Materials and methods: The patients presented with high intraocular pressure and blurred vision following a topiramate prescription for headache.

Conclusion: Timely recognition and intervention can prevent potential visual loss in such cases.

## Introduction

Several drugs are known to induce acute myopic shift in refraction along with acute attack of angle closure. The mechanism of causation is not clearly understood and several theories have been proposed. Topiramate is a commonly used antiepileptic drug for migraine prophylaxis. 

We reported a case series of topiramate-induced acute myopia and acute angle closure.

## Materials and methods

We reported two cases in the age group of 16-40 years, who presented to the emergency with sudden loss of vision with mild pain and redness. On obtaining the medical history, they were on tablet Topiramate 25 mg once daily for the last 7 days. We discussed the varied presentation and management of Topiramate-induced ocular complications in this case series.

## Results

*Case series*

*Case 1*

A 17-year-old boy presented with a history of sudden onset of painless decrease in vision in both eyes. His previous ocular records showed his visual acuity to be 20/20 and near vision N6 with emmetropic refraction. He was on topiramate 25 mg once daily for headaches for the past week. On examination, his visual acuity was 3/60 improving with pinhole to 20/20 and near vision was N6 close to face. Refraction revealed a value of -8.00 DS/-1.0 DC x 60° in the right eye and 9.00 DS/-1.0 DC x 90° in the left eye. The anterior chamber (AC) was shallow peripherally and intraocular pressure (IOP) measured by applanation was 35 mm of Hg in both eyes. Gonioscopy showed 360 degrees of appositional angle closure in both eyes, which opened on compression up to posterior trabecular meshwork. Examination of the fundus showed the presence of fine retinal striae radiating from the fovea. A diagnosis of topiramate-induced acute myopia with acute angle closure was made and the drug was discontinued immediately in consultation with the neurologist. He was also started on tablet acetazolamide 250 mg stat on 0.5% timolol topically for the reduction of IOP. 

When reviewed 48 hours after medication discontinuation, his unaided visual acuity improved to 20/20. AC was of normal depth, IOP measured by applanation was 14 mm of Hg and gonioscopy showed 360 degrees open angles in both eyes. Fundus examination was within normal limits with the disappearance of retinal striae. 

*Case 2*

A 40-year-old man presented to us with a history of sudden onset of bilateral painless diminution of vision. He has been using spectacles of -1.00DS/-1DC x 90° in the right eye and -1DS/-1.00DC x 90° in the left eye for the past 7 years. He was treated by the neurologist for headache and was on topiramate 25 mg once daily for the past 7 days. On examination, his visual acuity in both eyes was 2/200 improving with pinhole to 20/20, near vision N6. Refraction was -8.00 DS/ 1 DC x 90° in the right eye and -9.00DS /-1.00DC x 90° in the left eye. AC was of normal depth and IOP measured by applanation was 10 mm of Hg in both eyes. The gonioscopy showed 360-degree open angles in both eyes. Cycloplegic refraction was -5.00 DS/-1 DC x 90° in the right eye and -4.00 DS/-1.0 DC x 90° in the left eye. Examination of the fundus showed the presence of fine retinal striae radiating from the fovea and no peripheral choroidal detachment. A diagnosis of topiramate-induced acute myopia was made and the drug was discontinued immediately in consultation with the neurologist. When reviewed 48 hours after the discontinuation of medication, his BCVA was 20/20 with previous acceptance. AC was of normal depth and IOP measured by applanation was 12 mm of Hg. The gonioscopy showed 360-degree open angles in both eyes. Fundus examination was within normal limits with the disappearance of retinal striae. 

## Discussion

Topiramate is an oral sulfamate medication used primarily for seizure treatment that also demonstrates preliminary efficacy in the treatment of bipolar disorders and pain control of migraine.

The exact mechanism by which Topiramate induces myopia is not clearly understood. In the first case, myopia persisted despite cycloplegia hence accommodative spasm alone is unlikely to be the causative factor. Swelling of the crystalline lens due to the alterations in the sodium and chloride movement caused by the weak carbonic anhydrase inhibitor activity of topiramate has been postulated [**1**]. Prostaglandin-mediated ciliary body swelling causing forward movement of the crystalline lens has been documented using ultrasound biomicroscopy [**2**]. The second case was not associated with shallow AC or angle closure. A similar case of acute myopia in the absence of angle closure has been reported [**3**]. Retinal striae can result from altered membrane potential and are not the cause of myopia.

These cases will likely be misdiagnosed as bilateral angle closure glaucoma or accommodative spasm. Whenever a case of bilateral acute angle-closure glaucoma associated with myopia and a shallow anterior chamber is encountered, ciliochoroidal effusion syndrome induced by drugs should be considered in the differential diagnosis. As the mechanism of angle closure does not involve pupillary block peripheral iridotomy is ineffective in these cases [**2**]. 

The majority of reported adverse events have occurred in female patients (up to 89%) with a mean age of 36.5 years [**4**]. Both the cases mentioned above are in male patients of younger age.

With the increase in the frequency of prescribing topiramate for migraine prophylaxis, ophthalmologists and physicians must be aware of these complications [**5**].

## Conclusions

Early recognition and intervention can prevent potential visual loss in such cases. It is also important to educate the patients regarding the possible symptoms so that they can report at the earliest.

**Conflict of Interest Statement**

The authors state no conflict of interest.

**Informed Consent and Human and Animal Rights Statement**

Informed consent has been obtained from all individuals included in this study.

**Authorization for the use of human subjects**

Ethical approval: The research related to human use complies with all the relevant national regulations, and institutional policies, as per the tenets of the Helsinki Declaration, and has been approved by the review board of Father Muller Medical College, Mangalore, India (IEC/FMCH/023-61-2023).

**Acknowledgments**

None.

**Sources of Funding**

None.

**Disclosures**

Nil.
